# Ethnoecology of *miriti* (*Mauritia flexuosa,* L.f.) fruit extraction in the Brazilian Amazon: knowledge and practices of riverine peoples contribute to the biodiversity conservation

**DOI:** 10.1186/s13002-020-00430-z

**Published:** 2021-01-07

**Authors:** Flávio Bezerra Barros, Fagner Freires de Sousa, Josiele Pantoja de Andrade, Fabrício Menezes Ramos, Camila Vieira-da-Silva

**Affiliations:** 1 Federal University of Para, Belém, Brazil; 2 Federal Institute of Education, Science and Technology of Para–Campus Cametá, Cametá, Brazil; 3 Federal Institute of Education, Science and Technology of Para–Campus Vigia, Vigia, Brazil

**Keywords:** Agrobiodiversity, Floodplain forest, *Mauritia flexuosa*, Natural resources use, Nature and society relations

## Abstract

**Background:**

This article presents, from an ethnoecological perspective, the worldviews, traditional knowledge, and cultural practices of Amazonian riverine people involved in the extraction of *miriti* fruits (*Mauritia flexuosa* L.f.), in a context of increasing market demand for *miriti* fruits and of pressure for the intensification of *açaí* (*Euterpe oleracea* Mart.) production on the Sirituba island, in Abaetetuba, Brazil.

**Methods:**

Methods used were participant observation and non-directive interviews with 22 extractive families of miriti from the Santa Maria and Costa Sirituba communities, on Sirituba Island, in Abaetetuba, Pará, Brazil. Non-structured interviews were used to analyze the knowledge about the species, history of *miriti* extraction, the traditions, and innovations related to this activity over time. Participant observation took place when riverine individuals were working with *miriti* fruits, in order to grasp the “codes” that permeate the human-nature relationships embedded in this production system.

**Results:**

It was verified that the riverine peoples have a great knowledge about the palm tree, which is reflected in their own classification systems and management practices that allow the sustainable extraction of the fruits, avoiding, for example, cutting the *miriti* palms. In addition, a reciprocity relationship was observed between riverine peoples and *miriti* palm that are personified and preserved, contributing to the conservation of the species in the floodplain, even with the intensification of *açaí* (Euterpe oleracea Mart.) production. Another important aspect is the collective work involving all the members of the family, which allows the appropriation of the knowledge about the extraction of *miriti* by the young, allowing the resistance of the tradition that remains strong, contributing to the sustainability of the practice and conservation of biodiversity in the Amazonian floodplain.

**Conclusion:**

The k-c-p complex inherent to the riverine universe allows, even in face of the growing commercial demand for *miriti* fruits and the unchallenged increase in the extraction of this product, the conservation of floodplain biodiversity. Thus, we emphasize the importance of traditional knowledge and practices for biodiversity preservation, and they use them to guide public policies and natural resource management systems, aiming for sustainable ways to manage and use biodiversity.

## Introduction

The relationship between humans and nature has been studied by many scholars, especially in the field of ethnoecology, a science devoted to studying traditional knowledge or wisdom. Ethnoecology as a science is unique in that it seeks to grasp the non-formal knowledge forms—which Lévi-Strauss [1] defines as science of the concrete—in a systemic way, never dissociating beliefs (*kosmos*) from knowledge (*corpus*) and practices (*praxis*), the so-called K-C-P complex as described by Toledo and Barrera-Bassols [2, 3].

Traditional peoples and communities in Brazil are defined as culturally differentiated groups, who recognize themselves as such with their own forms of social organization; these groups occupy and use territories and natural resources as condition for their cultural, social, religious, ancestral, and economic reproduction, using knowledge, innovations, and practices generated and transmitted through tradition [4]. These groups have very old but equally valid form to understand and manage biodiversity in favor of nature preservation and biocultural reproduction because, as expressed by Toledo and Barrera-Bassols [3], “according to their worldviews, nature is the main source of life that feeds, supports, and teaches.”

Knowledge of nature among traditional peoples is especially noteworthy and reflects the depth and wealth of observations made by such groups within their sociocultural universes. This results in practices developed, improved, and transmitted over time [4, 5], reproducing and (re)constructing traditions that are dynamic in nature and adapted to conditions imposed by the environment or the surrounding society [6, 7].

The individuals devoted to *miriti* fruit (*Mauritia flexuosa* L.f.) extraction in the Amazon estuary name themselves as riverine peoples, who are the traditional peoples that inhabit riverbanks and predominantly make a living by managing natural resources from forests and waterways and by raising livestock [8–11]. They have complex systems of knowledge and representations emanating from the relationship established with natural resources.

We understand *miriti* fruit extraction practiced by riverine peoples as an interaction between humans (riverine peoples) and nature (especially *miriti* palm), mediated by empirical knowledge and symbolic elements capable of guiding riverine practices, constituting various management systems and, as a consequence, domestication of species [12–14]. The extraction, if conducted reciprocally, provides an opportunity for biodiversity conservation and the maintenance of livelihoods of the people who depend on this resource for their material and symbolic reproduction [15–17].

Thus, we question the analysis of the extraction by Homma [18] based on assumptions from neoclassical economics, which asserts that extractive activities are not sustainable since increasing market demand also increases pressure on natural resources, leading to over-exploitation and decline. Homma’s analyses separate human from nature, disregarding their traditional knowledge an ability to create strategies that allow for sustainable practices [19]. Proof of this is that even with growing demand for *miriti* fruits and its derivatives in Abaetetuba, the main marking place for these products, *miriti* fruit extraction is still a traditional practice, which is conducted in the family sphere, characterized by the use of simple technologies, and strongly influenced by riverine peoples’ traditional knowledge and beliefs [20].

In addition, on Sirituba Island, there is also pressure to intensify the production of *açaí* due to the increased demand for *açaí* fruit in recent decades and the great value attributed to the species. This has led research and rural extension institutions to advise the reduction of biodiversity for the benefit of *açaí* palm and instructions for management of the palm to bear fruit in winter, out of season. However, some people do not support these measures because they believe that “they are against the will of God”. Thus, species such as *miriti*, which riverine people are instructed to remove from the soil to make room for *açaí* trees, are preserved because, in addition to bearing fruit, the interlocutors believe contrary to external agents’ claims that this palm benefits *açaí* palms because its deep roots pull up water that keep soils moist and its large leaves generate organic matter fertilizing soils. Therefore, *açaí* growing around the *miriti* palms are healthier and bearing more fruits, which are meatier and more delicious. Thus, even with great external pressure for increasing the dominance of açaí trees among the riverine FPEs on the island, *miriti* trees have been preserved—albeit in less dense stands—and the tradition involving palm management remains strong, providing it with notoriety. Sirituba Island is recognized by those from other islands and the municipality of Abaetetuba as a major producer of *miriti* pulp; this fact was fundamental to the choice of the island as our research site.

The hypotheses that underpin this article are the following: (a) *miriti* fruit collectors possess a complex knowledge system regarding the palm tree, which allows sustainable forms of management and harvesting; (b) riverine beliefs guide their practices and establish a reciprocal relationship between humans and the palm tree; (c) traditional practices are dynamic, allowing the riverine to innovate and adapt to market requirements in a sustainable manner; (d) use of family labor allows for the exchange of knowledge and hence the sustainability of the tradition.

Thus, the objective of our paper is to present traditional knowledge and practices of *miriti* fruit collectors of the Sirituba Island, Amazon estuary, and analyze the sustainability of this traditional practice in light of environmental and economic tensions facing these groups in recent decades, with the main tension being the increasing demand for *miriti* fruit in town of Abaetetuba, PA. With this analysis, we aim to add to discussions on how the extraction of non-timber forest products by traditional peoples may contribute to sustainable development.

## Materials and methods

### Characterization of the study area

**Fig. 1:**
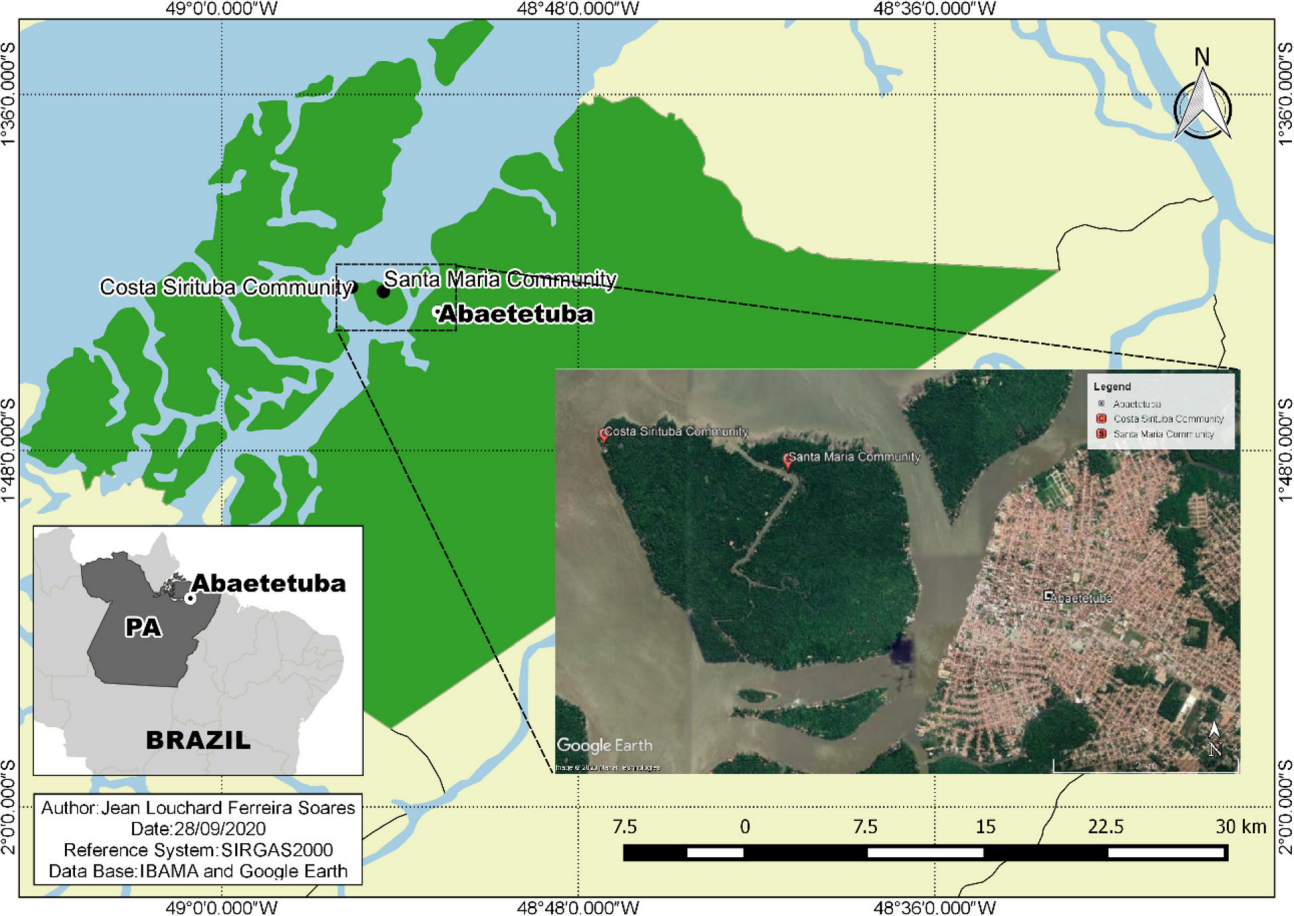
Location map of the municipality of Abaetetuba and Sirituba Island, Pará, Brazil

Sirituba Island (Fig. 1) is located in the Pará River, in the municipality of Abaetetuba, state of Pará, and it has a total area of 758,328.3 ha. The superintendency of Union Heritage in Pará state (SPU/PA) [21] manages the area, which is characterized as a floodplain area, because its lands are located on the alluvial floodplain, which emerges every day according to the tide regimes—high tide and low tide [22].

It consists of four traditional riverine communities: Costa Sirituba, Costa Campopema, Tabatinga, and Santa Maria do Rio Sirituba, whose ownership status was regularized in 2005, by granting Terms of Sustainable Use Authorization (TAUS) to the Our Floodplain Program of SPU/PA, and then the creation of the Agroextraction Settlement Project (PAE) “Santa Maria,” which benefited 248 families devoted to riverine agroextraction [23]. Out of the four communities, two practice *miriti* fruit extraction: Santa Maria and Costa Sirituba. Therefore, our research covered these two communities.

The community Santa Maria of Sirituba river, or simply Sirituba as it called by community residents, is located in the center of the island, with riverine houses situated on the banks of the Sirituba River. Sirituba Costa is located in the coastal part of the island, on the edge of Pará River at the confluence of the Bay of Capim. The houses are mostly of wood, built on stilts that extend across river and stream banks, forming conglomerates in some sections that characterize extensive households.

The formation of people who today make up the region of the Amazon floodplain dates from time immemorial. This process reflects the history of Amazon occupation, which is marked by the encounter of different cultures (indigenous, African, and European) that together gave rise to mestizo societies [24]. Thus, the riverine people are mestizos who have inherited in particular indigenous beliefs and thorough knowledge of terrestrial and aquatic environments, which characterize floodplain livelihoods. The riverine people who reside in the study communities today are mostly natives of the island or the costume of parents donating land parcels near to their homes for their children to establish his/her own household. Thus, there are significant kinship relations between the families [20].

The communities have elementary and high school facilities and school transport services by boat, which enables bringing children and young individuals from their homes to the school. At least once a month, the community receives doctors and dentists who provide health services and support for community health care workers [20].

Transportation in the communities is mainly by boat, especially small-sized ones equipped with a diesel engine, such as *rabetas* and *voadeiras* are used on a smaller scale. Throughout the day, there is continued movement of people who go to nearby towns, neighboring communities, or to visit friends or relatives within the community itself. Furthermore, boats also ensure the transport of forest and farm products and other consumer goods [20].

**Fig. 2:**
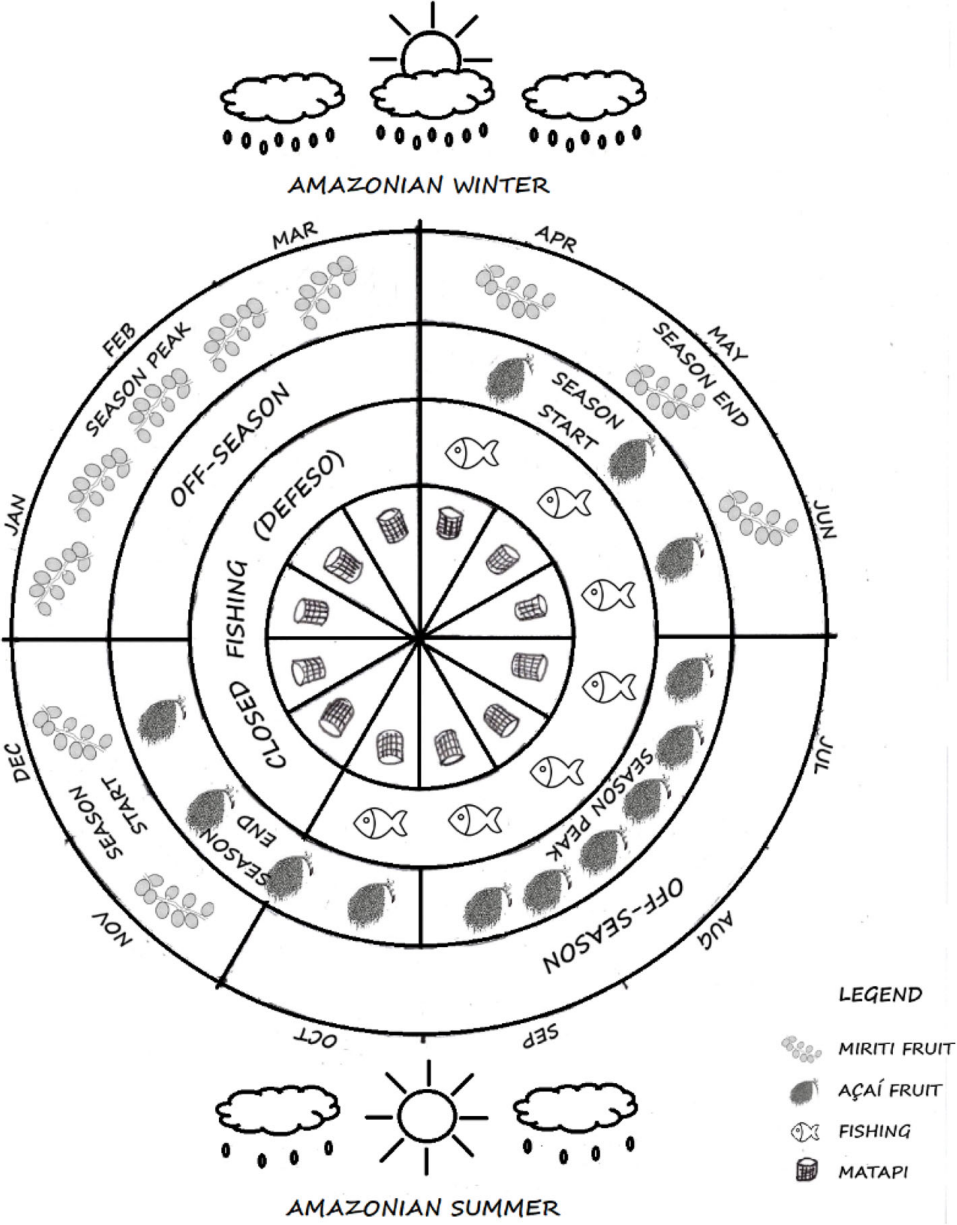
Production and extraction schedule maintained by riverine residents on Sirituba island, Abaetetuba, Pará, Brazil

Currently, the major economic activities are *açaí* fruit (*Euterpe oleracea* Mart.) cultivation and extraction, followed by the fabrication of *matapi* (a fishing apparatus produced through *jupati* splints, a *Rhapis excelsa* palm tree species), fishing and shrimp trapping, as well as extraction of seeds, fruits, and fibers, with an emphasis on *miriti* fruit extracted according to a specific calendar driven by the environmental conditions posed by the floodplain (Fig. 2).

The extraction of *miriti* on the island is considered a traditional practice integrated into riverine production systems since the onset of occupation. In this way, the practice is maintained by traditional knowledge, which is passed down from parent to child by way of oral traditions and work initiation processes. The *miriti* palms bear fruit in winter when *açaí* is not in season; therefore, they generate food and income for riverine people within a period that, due to scarcity of natural resources available in the floodplain, is referred the period of famiture [20]

### Data collection methods

The survey was conducted through fieldwork from August 2014 to May 2015, with an increased length of stay between January and May 2015, the *miriti* fruit season. Within this period, primary data collection was carried out using non-structured interviews [25] and participatory observation [26] with 22 riverine family *miriti* fruit extraction practitioners. The selection of families took into account the fact that they are actively working with the *miriti* fruit extraction for commercialization and consumption during the 2014/2015 season and the interest and availability to participate of research. The socioeconomic and cultural characteristics of these families are shown in the Table 1.

**Table 1:** Socioeconomic and cultural characteristics of *miriti* fruit extractors from Sirituba Islan, Abaetetuba, Brazil

**Categories**	**Variables**	**Results (%)**
**Interview information (head of household)**		
**Age**	16 to 20 years old	4.5
	21 to 25 years old	9.1
	26 to 30 years old	4.5
	36 to 40 years old	4.5
	41 to 45 years old	27.3
	46 to 50 years old	13.6
	more then 50 years old	36.4
**Sex**	Male	72.7
	Female	27.3
**Naturality**	Abaetetuba, PA, Brazil	100.0
**Marital status**	Single	18.2
	Married	54.5
	Common law marriage	22.7
	Widower	4.5
**Religion**	Catholic	90.9
	Evangelical	9.1
**Time that is extractor of** ***miriti*** **fruit**	From 1 to 5 years	4.5
	Over 20 years	95.5
**Who taught the practices related to** ***miriti*** **fruit extraction**	The parents	90.9
	Other family members	9.1
**Interviewee’s family information**		
**Family composition**	2 to 3 people	9.1
	4 to 5 people	36.4
	6 to 7 people	40.9
	8 to 9 people	4.5
	10 or more people	9.1
**Annual productive activities (excluding the** ***miriti*** **fruit extraction)**	Production of *açaí* fruit and fabrication of *matapi*	13.6
	Production of açaí fruit	9.1
	Production of *açaí* fruit and fishing	40.9
	Production of *açaí* fruit, fabrication of *matapi* and fishing	36.4
**Destination of** ***miriti*** **fruit**	Consumption and marketing	100.0
**Ways of marketing** ***miriti*** **fruit**	In natura	4.5
	Pulp	54.5
	Softened fruits (miriti mole) and pulp	40.9
**Marketing channels of** ***miriti*** **fruits**	Middlemen (Marreteiro)	4.5
	Extractivist-processors (in Sirituba Island)	4.5
	Processors (in Abaetetuba city)	40.9
	Consumers	31.8
	Processors (in Abaetetuba city) and consumers	13.6
	Middlemen, processors (in Abaetetuba city), and consumers	4.5
**Is miriti the main source of income in winter?**	Yes	86.4
	No	13.6
**Complementary sources of income**	Retirement pensions	18.2
	*Bolsa família* (a social welfare payment program)	36.4
	*Seguro defeso* (stipends paid to fishers during months when fishing is prohibited)	4.5
	*Bolsa Família*, *seguro defeso*, and *bolsa verde* (a payment for environmental services program)	9.1
	*Bolsa família* and fabrication of *matapi*	4.5
	*Bolsa família*, *bolsa verde*, retirement pensions and fabrication of *matapi*	4.5
	None	22.7

Non-structured interviews were used to analyze the history of *miriti* extraction, the traditions, and innovations related to this activity over time. Interviews provided us with the opportunity to engage in an open-ended dialogue with interlocutors, who expressed their ways of looking at and relating to nature. This allowed us to grasp elements indicative of their beliefs, knowledge, and practices that were derived from (and result in) their way of interacting with nature, in particular with *miriti* palms. Some guiding questions were used to conduct the interviews, both in relation to knowledge about the species (is there more than one type of *miriti*? If so, what are the differences between them? Are any of these preferred by the market? How do you identify that the *miriti* fruit is good to collect? What indicates that *miriti* fruit can already be processed? etc.) and how much in relation to the practice of extraction (how long have you practiced extraction of *miriti* fruit? With whom did you learn? Have there been changes in practices related to extraction throughout of the time? If so, what were these changes? Has the greater demand for *miriti* fruit in the market changed the way of collecting the fruits? Are young people in the family learning to work with *miriti* fruit extraction? etc.). All interviews were recorded, with prior authorization, and later transcribed.

Participant observation took place when riverine individuals were working with *miriti* fruits, in order to grasp the “codes” that permeate the human-nature relationships embedded in this production system. Thus, observations were made during fruit collection in the forest, fruit transportation to FPE’s, post-collection preparations (washing, pre-selection, “blackening”, selection), softening, making pulp, and trading, in order to describe and understand the activity, associated knowledge, and the transmission of such knowledge between generations. Records of observations were made in cameras and field notebooks for further analysis.

Collected data were systematized and analyzed after returning from the field, being interpreted in the light of the theory. The sustainability of the practices was considered based on the confrontation of the practices developed by the extractors of *miriti* from Sirituba Island, with those developed by other populations, as well as with the instructions of external agents, based on their k-c-p complex.

The ethical aspects of research with traditional peoples were considered, and the research was conditional upon authorization, by signing a statement of prior consent, by the chair of the Association of the Agroextrativist Settlement Santa Maria of Sirituba Island, legal representative of the residents of the communities where the research was carried out. The request for authorization to access traditional knowledge associated with genetic heritage was submitted to the National Historical and Artistic Heritage Institute - IPHAN, through process number 01450.012454/2014-00.

## Results and discussion

### Traditional knowledge of riverine peoples related to *miriti*

The knowledge of riverine people from Sirituba Island regarding floodplain species and particularly regarding the *miriti* palm are consistent with the idea advocated by Lévi-Strauss in Savage Minds [1]; therefore, we regard them as scientists herein. By interacting with the Amazonian floodplain, through observation, experimentation, and ancient knowledge, they have constructed a complex corpus, which allows them to produce and reproduce themselves in this environment using natural resources as foods, for curing diseases, and in building and obtaining an income that enables them to enjoy goods they themselves do not produce or obtain from nature.

The *miriti* palm deserves special attention in the context of the floodplains’ great diversity. This large palm tree, which sometimes plays a leading role in songs, soap operas, and travelers’ accounts [27], is regarded as a saint tree in the floodplain of this Amazonian region. Why do people assign holiness to a palm tree? The primary explanation for the holiness of the *miriti* palm is that it provides local society with “everything.” According to our informants, all parts are used for some purpose. Thus, the knowledge of *miriti* is great because, as we have already mentioned based on Lévi-Strauss [1], if a plant is regarded as useful, it also needs to be well-known.

Riverine peoples’ knowledge of the *miriti* tree is indeed vast, especially among the elderly, who had nature as their first (and sometimes the only) school. Only five decades ago on the island, understanding nature was necessary to become a “grown-up,” as interviewer 1, a 67-year-old riverine woman, stated. She was born and raised on the island and has known about the resources available on the floodplain since she was young.

Knowledge regarding the *miriti* tree involves botanical, environmental, and ecological aspects of the species. Observation makes it possible to identify flowering, fruiting, and fruit-ripening periods, as well as the time when the fruits are tastier:

The *miriti* fruit emerges in late summer [July to December]. From November onward, *miriti* fruits are already falling, and ripening (Interviewer 2, 64 years old, riverine man from Costa Sirituba).

In December, the old trees, those of an advanced age are good to be eaten, but new trees are not that ripe... good *miriti* fruits are found from January onwards, when they fall down (Interviewer 3, 82 years old, riverine man from Santa Maria).

According to the interlocutors’ observations, early *miriti* fruit ripening occurs in late summer, around November, when the fruits from some palm trees, mostly the older ones, begin to fall. However, it is believed that *miriti* fruits are delicious (good) from January onwards, extending to mid-May, a period defined as the “*miriti* fruit time”. The season closes between May and June, when the *açaí* fruit season begins.

*Miriti* is a dioecious species; hence, some palm trees only bloom and do not bear fruit, something which riverine individuals understand quite well. Something that caught our attention, however, was the distinction they assign to female palms, which are believed to have particular features—some of them more delicious fruits than others. When asked how such identifications took place, the first answers suggested fruit tasting and sensory distinction. Over time, other answers emerged, such as observing the habits of specific animals, such as *mucura* (*Didelphis marsupialis*) and chickens (*Gallus gallus domesticus*), which prefer fruits of the same palm trees even when others were present or more readily accessible. This corroborates once again the theory proposed by Lévi-Strauss [1] concerning the science of the concrete.

We already know the tree that is better, we know that *miriti* tree over there is good…because this happens, some *miriti* fruits just have no taste, they just look like the fruit... even *mucura*, a wild animal, they eat the best fruits, they prefer tastier *miriti* fruits. You can see where a lot of *miriti* fruit was eaten beneath the tree, you can take this *miriti* fruit, wash it, and eat it… it is good, yummy! An animal knows which is the best (Interviewer 4, 44 years old, riverine man from Santa Maria).

There is a tree here that we name as chickens’ *miriti* tree, because when the fruits fall down, the chickens immediately come to eat them, because they are very good. If we delay, none remains for us because the chickens eat everything (Interviewer 5, 56 years old, riverine woman from Costa Sirituba).

Palm trees, regarded as “good fruit providers,” are assigned names, constituting personification or anthropomorphization as described by Descola [15, 28]. From this point onwards, the human-*miriti* tree relationship intensifies because people start addressing the species in a special way, respecting and preserving it. Residents do not cut these palms’ bunches, and their fruits are not intended for trade, but rather reserved for family consumption or for gifting to friends and relatives. Their “children” are also preserved, which—as riverine individuals believe—will bear fruits as good as their “mother’s”. The mother’s fruits are also left for animals to feed upon because, as mentioned, animals prefer the best fruits.

### Ages, colors, shapes, and flavors: the riverine peoples’ ethnoclassification system

The close relationship between riverine individuals, the *miriti* tree, and their observations and insights about this palm allows them to identify specific attributes and properties of each tree. This makes it possible for them to find various qualities and assign different uses, a quality that according to Descola [15] is characteristic of traditional peoples; in this way, categories are established within a species through metaphorical and metonymic schemes that when organized permit the construction of a classification system. This comes close to the categories used by Berlin et al. [29]  and Berlin [29], in ethnobiology studies that mention several categories to compare the scientific and traditional peoples’ taxonomies, such as, for instance, morphology.

Riverine peoples’ categorization comprises both the palm tree (*miritizeiro*) and the *miriti* fruit (Table 2). The palm tree is categorized according to sex, as pointed out above, but also according to age (old and new) and size (large and small)—categories that have similar features since old trees are usually regarded as large and new trees are regarded as small. Thus, it is believed that old/large trees provide the sweetest fruits, while new/small produce bitter fruits, which will become sweeter as the palm tree grows and becomes older. For example, Interviewer 6, a riverine man from Santa Maria do Rio Sirituba reports, “the higher the *miritizeiro*, the better the *miriti* (...), it becomes sweet, *miriti* is sweet”.

**Table 2:** Ethnoclassification system of the miriti adopted by the riverine peoples inhabitants of the Sirituba Island, Abaetetuba, Brazil

	Cat.	Clas.	Characteristics
***Miriti*** **palm**	Sex	Male	Palm tree that does not bear fruit, just puts flowers. It is usually knocked down for port construction. However, riverine people dwellers emphasize that they only do so in cases of extreme need, as they recognize that female palms need the male to bear fruit.
		Female	Palm tree that bears fruit. Preserved for fruit collection.
	Age	Old	Palm trees with more than three decades, usually females that for the long time of fruiting are considered providers of good fruits, the “mothers” within the miritizal. They present greater representativeness in the riverine people sociocultural system, as they are attributed name to these palm trees, being respected by the families. Its fruits are special and, therefore, the bunches are not cut; it is only collected on the ground, restricting itself to family consumption and as a gift to friends, neighbors and family members who live in the city.
		New	Palm trees up to two decades old. In the case of females, their fruits are considered sour and are not very popular for family consumption, although they are collected for sale. When they are close to an old palm tree, they are conserved and cared for, as they are believed to be “daughters,” and when they reach the age of their “mothers,” they will offer fruit as good as them.
	Size	Large	Larger palm trees, which can be attributed to difficulty in climbing to cut the bunches in the period of fruit collection, in some of these no one dares to climb, thus, only take advantage of the fruit that fall. Sometimes, these palms are also considered ancient.
		Small	Small palm trees which are attributed to the ease of climbing to collect the fruits. They are also considered young.
***Miriti*** **fruit**	Color	White	Fruit which has clear or pale yellow pulp, tending to white. These fruitare are considered sweeter, better for pulp consumption, and good for preparing wine, although they do not present an attractive color. This factor is also attributed to the low demand for this type of fruit in the market, although it is mainly marketed to riverine people who are living in the city and know that it tastes better and orders it. Its occurrence is less frequent.
		Yellow	Fruit with yellow-orange pulp, slightly more intense than white. It presents a preference similar to white, also being referred to as sweet fruit and better for the consumption of the pulp and preparation of the wine, which presents better color and, therefore, is preferred for this form of use. It is more accepted in the market than white.
		Red	Fruit with purple-red pulp. They are generally referred to as sour and strong fruit, although they emerge as the most required by the market and, therefore, they are mainly aimed at commercialization
	Shape	Round	Circular-shaped fruit
		Long	Oval-shaped fruit
	Size	Big (*Graúdo*)	Big fruit
		Small (*Jitito*)	Small fruit
	Pulp ratio	Fleshy (*Carnudo*)	Fruits that present a high proportion of pulp in relation to the majority, resulting in greater yield when pulping for commercialization of the mass.
		Low flesh (*Seco*)	Fruits that have a low proportion of pulp in relation to the majority, resulting in low yield when pulping for commercialization of the mass.
	Collection method	Assembled (*Juntado*)	Fruits that are collected under the palm trees of miriti presenting ripe bunches. They are considered the fruit of the “right time” and, therefore, more delicious, “better to eat because it is very ripe”.
		Cut (*Cortado*)	Fruits obtained by the bunch cutting process. They are generally considered sour because they were harvested “ahead of time,” which implies that they are unripe. They are predominantly intended for commercialization.

Several distinctions are assigned to the fruits, but the most notable concerns pulp coloration; three different *miriti* fruit varieties are defined: white *miriti* fruit, which has clear or pale yellow pulp; yellow *miriti* fruit, with yellow-orange pulp; and red *miriti* fruit, with purple-red pulp. Notably, this classification form is also observed among traditional peoples, such as the Maijuna from Peru [30]. Other groups identify only two varieties, yellow and red [31], and white *miriti* fruit is categorized as yellow. Sometimes, different uses are assigned to these *miriti* fruit varieties.

Red *miriti*, for instance, is predominantly intended for trade because it is regarded attractive and thus is preferred by local market goers. This fact is also observed in Peru, where the red variety has the highest price in the market [30, 31]. There are already projects to produce and spread seedlings of this variety to ensure the preservation of the species and promote economic empowerment of local communities [30].

Despite the commercial preference for red *miriti* fruit, as reported by Delgado et al. [31] and Gilmore et al. [30] in Peru and in Sirituba, white and yellow *miriti* fruits are also “harvested” and sold mainly in the form of a mixture (pulp and peel), due to the possibility of mixing the pulp of different varieties. However, we notice that these varieties as a rule are regarded as the tastiest and, as a consequence, are greatly appreciated by local families as a part of their diet. White *miriti* fruit is regarded as sweeter, and people prefer to eat it raw and fresh together with cassava flour. Yellow *miriti* fruit, which also has a mild taste, is eaten fresh; however, most extract wine [juice] to prepare porridge consumed on a daily basis in the morning when the fruit is in season.

People also regard *miriti* fruits as sweeter or sourer between fruits that are “assembled” and “cut,” categories related to collection processes. Assembled *miriti* fruit is that which is collected beneath palm trees after they ripen and spontaneously fall; these are the so-called “right time” fruits that are regarded as sweeter and of better quality. Cut *miriti* fruit, in turn, is that which is obtained by cutting bunches, occurring when the first fruits fall down. Thus, the fruits are not fully ripe; as a consequence, they are regarded as sour or tasteless. Similar findings were reported by Mota et al. [32] among mangaba fruit pickers from northeastern Brazil and by Vieira-da-Silva and Reis [33] among pine nut gatherers from southern Brazil.

Ah, the tastiest ones just fall down, those are pretty ripe. The cut ones, people often cut ahead of time (...), the fruit is still sour; when it is not really ripe, it is sour - its mixture, even its wine is sour ... and the fruit itself, we cannot even eat it [raw], when it is cut (Interviewer 3, an 82 year old riverine man from Santa Maria).

Assembled fruit is better to eat; it is pretty ripe; cut fruit is not, the one cuts and leavesit “blackening” for eight days... assembled fruit is different, one chooses it from beneath the tree; it is better, it is already ripe (Interviewer 7, a 63 years old, riverine man from Costa Sirituba).

Riverine people associate fruit ripening with peel coloration, a fact that allows identifying ripe fruit simply by looking at it. They report that ripe fruits have the darkest peel, black, or opaque red (“*tuíra*,” the local term). Thus, they stress that “assembled” fruits may be eaten up to 24 h after collection since they fall down already blackened, while “cut” fruits have to be left to “blacken” within a period from 5 to 7 days. During this time, the fruits are arranged on wooden and straw floors in huts built to accommodate *miriti*, the so-called “*miriti* fruit houses”. Afterwards, the climacteric fruit becomes ripe. This is also confirmed by the fact that the stem detaches from the fruits.

Insights in this regard were also reported by Mota et al. [32] among *mangaba* fruit pickers from northeastern Brazil who identify fruit ripening by peel color and classify mangaba fruits according to collection procedures, as being “windfall *mangaba* fruit”—those which fall down spontaneously—and “capsized *mangaba* fruit”, those “suddenly” collected (still unripe) and left to ripe, similarly to *miriti* fruit.

Knowledge of the ecology of the palm tree allows the riverine to define the “good” fruits to eat and/or sell, as well as to determine the period and the appropriate form of collection. According to Smith et al. [34], the local knowledge is important to make decisions about the use of biodiversity and its management. Schmidt and Ticktin [17] add that the ecological knowledge systems of traditional peoples on NWFPs are essential in the definition of sustainable management systems, contributing to the conservation of biodiversity.

### The tradition that reinvents itself: changes and innovations in *miriti* fruit extraction

For those who extract *miriti* fruit, the activity is viewed as a tradition; gatherers refer to past generations as having knowledge and practices that are passed on to succeeding generations [5], as observed in the narratives of some interlocutors.

This is very old... it is a tradition from our great-grandfathers, grandfathers, fathers, mothers. Something from a long time ago...when I realized I was a person the world, I was already working with *miriti* fruit (Interviewer 1, a 67 year old, riverine woman from Santa Maria).

I have worked with *miriti* since I realized I was a person in the world because my parents worked with it, with the fruit... since childhood I have worked with *miriti* (Interviewer 8, a 42 year old riverine man from Santa Maria).

I started working with *miriti* since my father’s time, right? First, people worked with *miriti* itself, right? Then, I started selling *miriti* fruit... and we were already participating in my father’s trade, right? (...) He died and we have always worked with *miriti* fruit. (...) My son has already taken control of the business (Interviewer 9, a 42 year old riverine woman from Santa Maria).

The above comments, although failing in temporal precision, enable us to affirm that *miriti* fruit extraction was already widely practiced in the past on the island. Fruit extraction was one of man activity practices undertaken by riverine families to be passed on from parents to children through teaching and daily practice. This corroborates the research of Toledo and Barrera-Bassols [3] who state that traditional knowledge is derived from the experience of subjects who are responsible for constructing such knowledge.

Tradition appropriated by riverine people when they are young is subsequently reproduced when they get married and establish new households. However, we emphasize that practices learned are subject to modifications and, in the case of *miriti* fruit extraction, have undergone changes mainly due to the introduction of new technologies and attributed to demands and/or consumer market requirements. This reflect the dynamic nature of traditional knowledge, which as claimed by Almeida [35] “sticks to actual processes and social subjects who dialectically transform their practices (...), redefining their social relationship with nature,” in order to ensure social representation [6].

### “At some point long ago they started cutting the bunches”: changes in the human-*miriti* relationship

Commercial interest in *miriti* fruits in the Abaetetuba region has led to an increased extraction of this resource, which is no longer simply “assembled” beneath palm trees; given the irregular fall of ripe fruits on a spontaneous basis, requiring greater time is required to collect the amount demanded by the market. This has occurred in indigenous communities from the Peruvian Amazon as well [30, 31], also causing changes in how the fruit is obtained. Almeida [35] refers to this as a redefinition of the man-nature relationship.

**Fig. 3:**
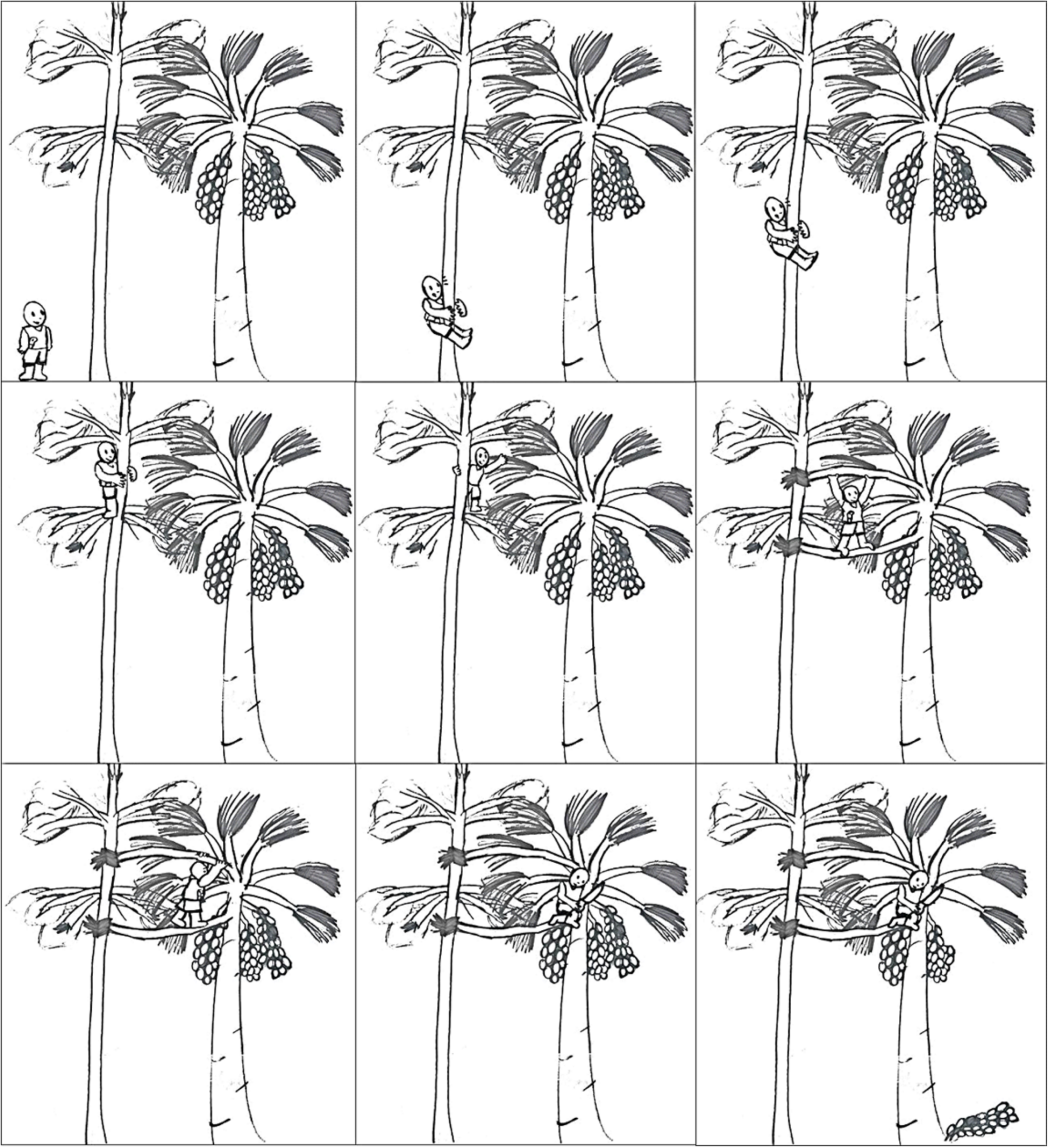
Climbing scheme to cut *miriti* tree bunches on Sirituba Island

Moreover, unlike Peruvian and Brazilian indigenous people who have come to cut palm trees in order to have access to fruits [30, 31, 36], riverine individuals from Sirituba began to cut only those bunches identified as ripe; this practice enables them to meet market needs within a required time period. This practice is also distinct from that adopted to collect açaí fruit (i.e., riverine individuals climb the palm tree to extract fruits). In the case of the *miriti* palm, people climb another tree close to the palm tree; then, a leaf from the latter is reached, which is pulled and tied to the canopy of the first, providing a bridge that enables the “cutter” to arrive at the *miriti* tree canopy, where the bunch is finally cut (Fig. 3). This is a strenuous activity that requires great skill and is a male activity.

This “new” way of relating to nature raises some discussions regarding the social group under study. Some fruit collectors believe that the new way of collecting does *not* undermine nature’s time and by extension therefore, God’s will [37]; the logic being that since cutting is limited to bunches where fruits “are already falling down” (i.e., they are ripe), they are collected at the “right time.” Others, usually collectors gathering fruits for household consumption, do not agree with this mindset; according to this group, fruits are picked “ahead of time”; thus, “for some time, *miriti* fruits do not grow every year”. In this regard, several ethnographic studies, such as Lima [38] conducted with the Katukina people, highlight the punishments that supernatural entities bring upon those who do not observe the natural order of things. Both social groups consider the possibility that nature fails to protect those who do not observe its rules.

According to Woortmann [37], residents’ concern with observing nature’s time or the time when “it can or wants to provide” fruits reflects moral principles of the human-nature relationship, where the former respects the latter so it is able to reciprocate. In this context, the author emphasizes the existence of a third *agent*, God, who is regarded as the “owner” of nature and, in this case, rewards man for respecting nature. Disrespect, on the other hand, is seen as an offense to God’s will, and as a consequence brings divine punishment, leading one’s family to the state of “precisão” [need]. Those who believe that cutting goes against God’s will illustrate this view in that the attribute the year that *miriti* fruits fail to grow with a time of need.

Our observations, however, allow us to show that collectors have a predominantly reciprocal relationship [15, 39] with species in the floodplain ecosystem and, above all, with the *miriti* tree. As previously mentioned, the palm is often personified, and people assign a symbolic value to it that prevents specific palms trees from being felled. This corroborates Cottee-Jones and Whittaker [40] who discuss the allocation of cultural values to certain species as a key to ensuring their preservation.

This reciprocity involving riverine people and the *miriti* tree has an ecological aspect that also benefits other species, such as animals that feed on *miriti* fruits, plant species that are favored by the shade of this palm, and the fertilizer produced through by decomposing leaves. Thus, by ensuring perpetuation of the *miriti* tree and the other plant species, the riverine individual also benefits, and her/his reproduction is guaranteed as a part of nature, corroborating the idea of Descola [15, 39] that relates the *kosmos*’ balance of reciprocal exchanges between beings of nature.

Moreover, we highlight riverine peoples’ ecological knowledge, flexibility, and resilience even under external pressure (from the market and research and rural outreach institutions advocating for production innovations). They have outlined strategies to ensure the reproduction of palm trees and, as a consequence, the maintenance of their way of life [6] because the practice of cutting fruits is a sustainable practice, satisfying the demand and preserving wild populations [41]. In Peru, climbing courses have been carried out to learn strategies of collecting miriti fruits in order to avoid felling palm trees [30, 31, 42, 43].

### New requirements, new knowledge: new tradition?

Increased demand for *miriti* fruits in the market of Abaetetuba, resulting from increased commercial interest in this product since the 1990s, implied changes not only in collection procedures, but also in certain practices and the incorporation of new instruments to process fruits that are derived from market requirements.

Transformations and the inclusion of new technologies into traditional practices to meet production (quantity and quality) demands of dominant markets [44] have been reported by other studies on various traditional groups, such as indigenous communities devoted to *miriti* fruit collection in Peru [30], pine nut gatherers from Santa Catarina and Rio Grande do Sul, southern Brazil [14, 45], and mangaba fruit pickers from northern and northeastern Brazil [32].

The results of these studies point out that such requirements have increased according to the scale achieved by these products that may range from local to global. However, none of them suggests that such requirements lead to the extinction of tradition; however, some authors outline the risks of biocultural erosion [46]. Traditional peoples have a degree of autonomy [47], and as a consequence, they find ways of meeting new requirements without denying their lifestyles. Moreover, they are observers and experimenters, introducing or rejecting “innovations” according to their perceptions [37, 48].

Thus, the *miriti* fruit collectors have stopped using *potes* (clay pot, commonly used to store water) and *tinas* (clay bowl) made of clay, which were adopted as containers to carry the fruits to fires for softening, and replaced them with metal cylinders (the so-called *latão* or *bidão*, in the local language) an object that has been used for about 20 years, as reported by Interviewer 10:

When I was born, I saw my mother softening *miriti* fruits in a *pote*, sometimes in a *tina*, big *tinas* made of clay. Using cylinders is a relatively recent procedure... I think it has been used for about 20 years; people have been using cylinders for about this length of time. I personally believe that the reason was that the cylinder can store more fruit... 12 *rasas*... Some pots cannot even store 1 *rasa*... So, it is not much to sell, and people have to work more (Interviewer 10, a 56 year old, riverine woman from Santa Maria).

The statement by Interviewer 10, a woman devoted to *miriti* fruit extraction since her childhood, reveals that the introduction of cylinders was due to cylinder capacity, which allows softening a larger amount of fruits per day. This provides a convenience for *miriti* collectors, who can process a greater amount of the product and thus meet market demand in a shorter amount of time while working less. Therefore, we notice that riverine people are not obtuse or against change; they are open to “innovation,” provided that it ensures family reproduction [37].

The same may be noticed regarding the adoption of “scraping”, which, due to market changes (i.e., demand for fruit mixture), came to be incorporated into riverine peoples’ practices for nearly 10 years as illustrated below:

Back then, at the time when my mother softened *miriti* fruits, and even I softened them a while ago, right? (...) We took everything in seed, the fruit, to it resell in the town, right? And recently, what are we doing? For about 10 years, people started scraping the *miriti* fruit (Interviewer 10, a 56 year old riverine woman from Santa Maria).

Previously, as reported by Interviewer 10, the fruits were only softened and sold as lumps; now, they are pulped, something, which, at that time, required new instruments. Thus, along with this new practice, *miriti* fruit “scraping,” some objects already observed in collectors’ sociocultural universe were taken from their original contexts to be used as working tools. These took on other functions and meanings: spoons became fruit scrapers; bowls and pots were used to store the pulp; and panniers, previously used to collect fruits, gained a new function: storing the soft fruits that were scraped.

**Fig. 4:**
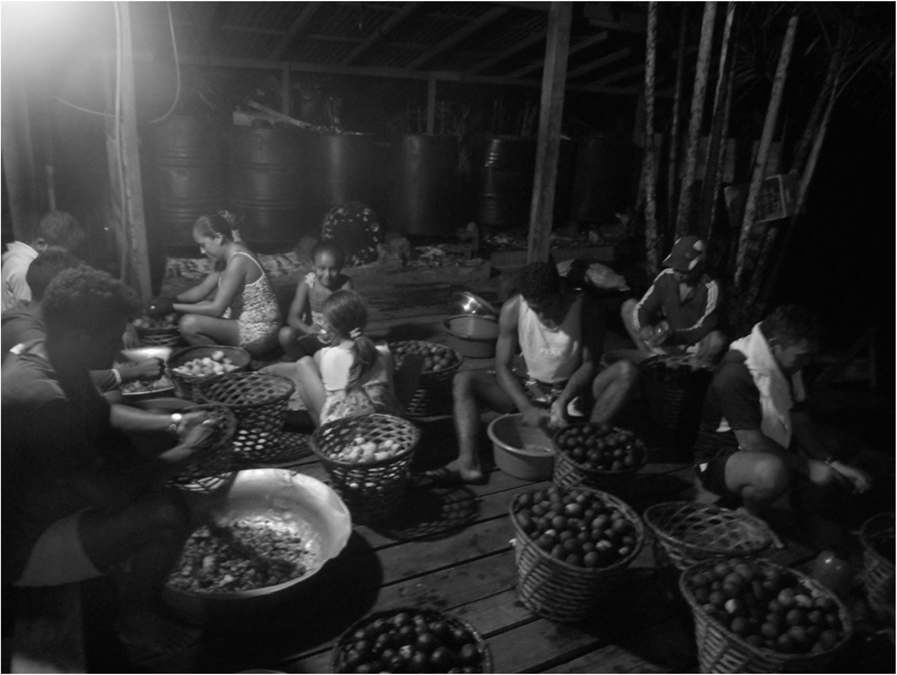
Riverine family scraping *miriti* fruits

The incorporation of scraping as a new practice also resulted in a rearrangement of FPE’s—as it required a greater availability of manpower and “time” to execute work. Thus, riverine individuals started to pulp fruits at dawn, a time at which family members—those who live in the same house and others who have a consanguineous or emotional connection [49]—gather for fulfilling the task (Fig. 4). Men, women, and children, armed with spoons and pots or bowls, sit on the ground or on improvised seats (stools) with soft *miriti* fruit rasas before them and remove the mass from *miriti* fruits. Their ability to fulfill this task is impressive. Adults and children quickly “scrape” fruits, which are carefully selected, separating those regarded as inadequate for trade or for consumption due to injury, rotting, or “slime”.

This period is often tiresome, and the work considered difficult, but it is also a time to talk about family affairs, the community, soap operas, and daily news. Stories, tales, and jokes are told—all of which ensures sociability [50] between riverine individuals, guaranteeing the (re)production of knowledge and practices transmitted through the interaction between various generations, (re)constructing such traditions on a collective basis.

This shows that the miriti extractivism practiced by the riverine people of Sirituba goes beyond a simple economic activity focused on the provision of the family; it is a traditional practice permeated with cultural values, which is performed independently of the income, corroborating with that found by Sakai et al. [51] about the use of non-timber forest products by people in rural Borneo.

## Conclusion

Knowledge, practices, and beliefs inherent to the riverine universe are clearly seen in the relationship Amazonian peasants (riverine peoples) have with the *miriti* tree—by personifying it, assigning holiness to it, and establishing a reciprocal pact. This allows, even in face of the growing commercial demand for *miriti* fruits (mixture) and the unchallenged increase in the extraction of this product, the conservation of floodplain biodiversity, and as a consequence, the reproduction of riverine people integral to the local ecosystem.

Similarly, we believe that the incorporation of new tools, practices, and above all, knowledge of *miriti* fruit extraction, resulting from external requirements of a dominant society, in the meaning proposed by Wolf [45], expresses the dynamic nature of tradition that, as pointed out by many scholars, adapts to conditions imposed from the outside to ensure group social reproduction. And, particularly in this case, we emphasize the production of a new sociability that, by interconnecting various riverine generations, ensures not only the transmission, but the joint construction of this tradition; we perceive this as the continued renewal of the past into the present.

Finally, we emphasize the importance of traditional knowledge and practices for biodiversity preservation and corroborate the numerous studies pointing to the need to recognize and use them to guide public policies and natural resource management systems, aiming for sustainable ways to manage and use biodiversity.

## Data Availability

All data have already been included in the manuscript. We are willing to share the data generated and analyzed during the current study.

## References

[1] Lévi-Strauss C (1989). O Pensamento Selvagem, 9th edição.

[2] Toledo VMM, Barrera-Bassols N (2009). A etnoecologia: uma ciência pós-normal que estuda as sabedorias tradicionais. Desenvolvimento e Meio Ambiente.

[3] Toledo VMM, Barrera-Bassols N (2015). A memória biocultural: a importância etnoecológica das sabedorias tradicionais, 1th edição.

[4] Brasil Casa Civil Decreto N° 6.040, de 7 de Fevereiro de 2007 (2007). Institui a Política Nacional de Desenvolvimento Sustentável dos Povos e Comunidades Tradicionais.

[5] Almeida MC (2010). Complexidade, saberes científicos, saberes da tradição.

[6] Harris M, Adams C, Murrieta R, Neves W (2006). Presente ambivalente: uma maneira amazônica de estar no tempo. Sociedades caboclas amazônicas: modernidade e invisibilidade.

[7] Santilli J (2005). Socioambientalismo e novos direitos: proteção jurídica à diversidade biológica e cultural.

[8] Hiraoka M, Furtado L, Leitão W, Mello AF (1993). Mudanças nos padrões econômicos de uma população ribeirinha do estuário do Amazonas. Povos das águas: realidade e perspectivas na Amazônia.

[9] Maybury-Lewis B, Furtado L (1997). Terra e água: identidade camponesa como referência de organização política entre ribeirinhos do rio Solimões. Amazônia: desenvolvimento, sociodiversidade e qualidade de vida.

[10] Fraxe TJP (2000). Homens anfíbios: etnografia de um campesinato das águas.

[11] Witkosky AC (2007). Terras, florestas e águas de trabalho: camponeses amazônicos e as formas de uso de seus recursos naturais.

[12] Clement CR (1999). 1492 and the loss of Amazonian crop genetic resources. I. The relation between domestication and human population decline. Econ Bot.

[13] Marinho JAM, Godoi EP, Menezes MA, Marin RA (2009). Desenvolvimento do extrativismo do açaí e mudança na socioeconomia de ribeirinhos marajoaras. Diversidade do campesinato: expressões e categorias.

[14] Vieira-da-Silva C, Miguel LA (2014). Extrativismo e Abordagem Sistêmica. Novos Cadernos NAEA.

[15] Descola P, Descola P, Pálsson G (2004). Constructing natures: symbolic ecology and social practice. Nature and society: anthropological perspectives New York.

[16] Arnold JEM, Pérez MR, Wollenberg E, Ingles A (1998). The role of non-timber forest products in conservation and development. Incomes from the forest: methods for the development and conservation of forest products for local communities.

[17] Schmidt IB, Ticktin T (2012). When lessons from population models and local ecological knowledge coincide – effects of flower stalk harvesting in the Brazilian savanna. Biol Conserv.

[18] Homma AKO (1990). A dinâmica do extrativismo vegetal na Amazônia: uma interpretação teórica.

[19] Rego JF (1999). Amazônia: do extrativismo ao neoextrativismo. Ciência Hoje..

[20] Sousa FF, Vieira-da-Silva C, Barros FB (2018). The (in)visible market of *miriti* (*Mauritia flexuosa* L.f.) fruits, the “winter acai”, in Amazonian riverine communities of Abaetetuba, Northern Brazil. Global Ecol Conserv.

[21] Instituto de Desenvolvimento Econômico, Social e Ambiental do Pará – IDESP (2011). Abaetetuba: estatística municipal.

[22] Silva LGT, Silva BNR, Rodrigues TE (2002). Análise fisiográfica das várzeas do Baixo Tocantins: uma contribuição ao manejo e desenvolvimento dos sistemas de uso da terra.

[23] Brasil INCRA/MDA (2005). Portaria n° 47, de 28 de novembro de 2005.

[24] Fraxe TJP, Witkoski AC, Pereira HS (2007). Comunidades ribeirinhas amazônicas: memória, ethos e identidade.

[25] Michelat G, Thiolent M (1987). Sobre a utilização de entrevista não diretiva em sociologia. Crítica metodológica, investigação social e enquete operária.

[26] Malinowski B (1976). Argonautas do Pacífico Ocidental: um relato do empreendimento e da aventura dos nativos nos arquipélagos da Nova Guiné Melanésia.

[27] Rosa JG (1976). Noites do sertão Rio de Janeiro.

[28] Descola P (1986). La nature domestique: symbolism et práxis dans l’écologie des Achuar.

[29] Sakai S, Choy YK, Kishimoto-Yamada K, Takano KT, Ichikawa M, Samejima H, Kato Y, Soda R, Ushio M, Saizen I, Nakashizuka T, Itioka T (2016). Social and ecological factors associated with the use of non-timber forest products by people in rural Borneo. Biol Conserv.

[30] Berlin B, Breedlove DE, Raven PH (1973). General principles of classifications and nomenclature in folk biology. Am J Phys Anthropol..

[31] Berlin B (1992). Ethnobiological classification: principles of categorization of plants and animals in traditional societies.

[32] Gilmore MP, Endress BA, Horn CM. The socio-cultural of *Mauritia flexuosa* palm swamps (aguajes) and implications for multi-use management in two Maijuna communities of the Peruvian Amazon. J Ethnobiol Ethnomed. 2013:9–29. 10.1186/1746-4269-9-29. 10.1186/1746-4269-9-29 PMC3733440 23607601

[33] Delgado C, Couturier G, Mejia K. *Mauritia flexuosa* (Arecaceae: Calamoideae), an Amazonian palm with cultivation purposes in Peru. Fruits; 2007;62:157-169. doi: 10.1051/fruits:2007011.

[34] Mota DM, Silva Júnior JF, Schmitz H, Rodrigues RFA (2011). A mangaba, as catadoras, o extrativismo.

[35] Vieira-da-Silva C, Reis MS (2009). Brazilian pine nuts’ production in Caçador’s region, SC: aspects of the attainment and its importance for local communities. Revista Árvore.

[36] Smith BM, Chakrabarti P, Chatterjee A, Chatterjee S, Dey UK, Dicks LV, Giri B, Laha S, Majhi RK, Basu P (2017). Collating and validating indigenous and local knowledge to apply multiple knowledge systems to an environmental challenge: a case-study of pollinators in India. Biol Conserv.

[37] Almeida AWB, Viegas DP, Buriol F (2014). Prefácio. Resistência das comunidades através da tradição.

[38] Ribeiro AH (2010). O *miriti* (*Mauritia flexuosa* L.f.) na terra indígena Araçá, Roraima: usos tradicionais, manejo e potencial produtivo.

[39] Woortmann EF, Godoi EP, Menezes MA, Marin RA (2009). O saber camponês: práticas ecológicas tradicionais e inovações. Diversidade do campesinato: expressões e categorias, v.2, São Paulo, SP: Editora UNESP/ Brasília, DF: Núcleo de Estudos Agrários e Desenvolvimento Rural.

[40] Lima EC (2008). Cobras, xamãs e caçadores entre os Katukina (pano). Tellus..

[41] Descola P, Kuper A (1992). Societies of nature and nature of society. Conceptualizing society.

[42] Cottee-Jones HEW, Whittaker RJ (2015). Felling Ficus: the cultural status of fig trees in a rural assamese community, India. Ethnobiology Lett..

[43] Isaza C, Bernal R, Galeano G, Martorell C (2017). Demography of Euterpe precatoria and Mauritia flexuosa in the Amazon: application of integral projection models for their harvest. Biotropica.

[44] Manzi M, Coomes OT (2009). Managing Amazonian palms for community use: a case of aguaje palm (*Mauritia flexuosa*) in Peru. Forest Ecol Manag.

[45] Holm JA, Miller CJ, Cropper WP (2008). Population dynamics of the dioecious Amazonian palm *Mauritia flexuosa*: simulation analysis of sustainable harvesting. Biotropica.

[46] Wolf E (1976). Sociedades camponesas.

[47] Vieira-da-Silva C, Miguel LA (2017). Marketing channel of pine nut and its agents in São Francisco de Paula - RS. Floresta.

[48] Shiva V (2003). Monoculturas da mente: perspectivas da biodiversidade e biotecnologia.

[49] Wanderley MNB, Tedesco JC (1999). Raízes históricas do campesinato brasileiro. Agricultura familiar: realidades e perspectivas.

[50] Sousa FF, Andrade JP, Kato OR (2014). Diversificação da produção e transição agroecológica: uma experiência com SAF na várzea do Rio Capim - PA. Cadernos de Agroecologia..

[51] Segalen M (1996). Sociologia da família.

[52] Mota DM (2005). Trabalho e sociabilidade em espaços rurais.

